# Fluorine-19 Magnetic Resonance Imaging for Detection of Amyloid β Oligomers Using a Keto Form of Curcumin Derivative in a Mouse Model of Alzheimer’s Disease

**DOI:** 10.3390/molecules26051362

**Published:** 2021-03-04

**Authors:** Daijiro Yanagisawa, Nor Faeizah Ibrahim, Hiroyasu Taguchi, Shigehiro Morikawa, Takami Tomiyama, Ikuo Tooyama

**Affiliations:** 1 Molecular Neuroscience Research Center, Shiga University of Medical Science, Seta Tsukinowa-cho, Otsu 520-2192, Japan; nor.faeizah.ibrahim@ppukm.ukm.edu.my (N.F.I.); taguti@belle.shiga-med.ac.jp (H.T.); morikawa@belle.shiga-med.ac.jp (S.M.); 2 Department of Biochemistry, Faculty of Medicine, Universiti Kebangsaan Malaysia Medical Centre, Jalan Yaacob Latif, Cheras 56000, Kuala Lumpur, Malaysia; 3 Department of Translational Neuroscience, Osaka City University Graduate School of Medicine, 1-4-3 Asahi-machi, Abeno-ku, Osaka 545-8585, Japan; tomi@med.osaka-cu.ac.jp

**Keywords:** magnetic resonance imaging, Alzheimer’s disease, mouse model, curcumin, keto-enol tautomerism, imaging biomarker

## Abstract

Recent evidence suggests that the formation of soluble amyloid β (Aβ) aggregates with high toxicity, such as oligomers and protofibrils, is a key event that causes Alzheimer’s disease (AD). However, understanding the pathophysiological role of such soluble Aβ aggregates in the brain in vivo could be difficult due to the lack of a clinically available method to detect, visualize, and quantify soluble Aβ aggregates in the brain. We had synthesized a novel fluorinated curcumin derivative with a fixed keto form, named as Shiga-Y51, which exhibited high selectivity to Aβ oligomers in vitro. In this study, we investigated the in vivo detection of Aβ oligomers by fluorine-19 (^19^F) magnetic resonance imaging (MRI) using Shiga-Y51 in an APP/PS1 double transgenic mouse model of AD. Significantly high levels of ^19^F signals were detected in the upper forebrain region of APP/PS1 mice compared with wild-type mice. Moreover, the highest levels of Aβ oligomers were detected in the upper forebrain region of APP/PS1 mice in enzyme-linked immunosorbent assay. These findings suggested that ^19^F-MRI using Shiga-Y51 detected Aβ oligomers in the in vivo brain. Therefore, ^19^F-MRI using Shiga-Y51 with a 7 T MR scanner could be a powerful tool for imaging Aβ oligomers in the brain.

## 1. Introduction

Amyloid β (Aβ), which is generally and ubiquitously expressed as peptides of primarily 40 and 42 residues, forms insoluble fibrils and accumulates in extracellular deposits known as senile plaques in the brain of patients with Alzheimer’s disease (AD) [1]. Genetic evidence in familial AD cases strongly supports the amyloid cascade hypothesis; that is, an imbalance between the production and clearance of Aβ is a very early, often initiating factor in AD [2,3,4]. Moreover, recent evidence further suggests that the formation of soluble Aβ aggregates with high toxicity, such as oligomers and protofibrils, is a key event causing AD [5,6,7,8,9,10]. However, understanding the pathophysiological role of such soluble Aβ aggregates in the brain in vivo could be difficult due to the lack of a clinically available method to detect, visualize, and quantify soluble Aβ aggregates in the brain.

**Figure 1:**
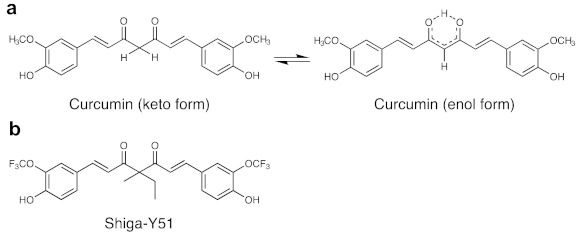
Chemical structures of curcumin and Shiga-Y51. (**a**) Curcumin, a yellow-orange pigment in turmeric, exists in an equilibrium between keto and enol tautomers. (**b**) Shiga-Y51 is a fluorinated curcumin derivative with a fixed keto form by the methyl and ethyl groups at the C4 position.

Recent advances in imaging techniques for in vivo detection of amyloid pathology in the brain have attracted much attention [11,12,13,14,15,16]. Several recent studies have reported about near-infrared (NIR) fluorescence imaging probes that selectively labeled Aβ oligomers in the in vivo brain of a mouse model of AD, including BoDipy-Oligomer (BD-Oligo) [17], F-SLOH [18], CRANAD-102 [19], PTO-29 [20], and DCM-AN [21]. We have also reported about a novel fluorinated curcumin derivative with high selectivity to Aβ oligomers, named as Shiga-Y51 [22]. Curcumin is a yellow-orange pigment in turmeric, which exists in an equilibrium state between the keto and enol tautomers (Figure 1), while Shiga-Y51 exists only in the keto form due to the methyl and ethyl groups at the C4 position. In our previous study, curcumin and the derivatives with keto-enol tautomerism were found to bind to Aβ fibrils primarily in the enol form, but the keto form of curcumin derivative cannot bind to Aβ fibrils [23]. Another study showed that curcumin and the derivatives with keto-enol tautomerism can bind to Aβ oligomers [24]. The compounds that can bind to Aβ fibrils, including thioflavin, Pittsburgh compound B (PiB), and the enol form of curcumin, have a common flat and rather rigid structure with a ring system that is co-planar and possesses π-electrons that are delocalized in the rings and move freely within them. We therefore hypothesized that these molecules with flat and rather rigid structures bind to Aβ fibrils easily but have some difficulty entering the hydrophobic core of Aβ oligomers because of their rigidity. However, suitably sized hydrophobic molecules with flexible structures, such as the keto form of curcumin derivative Shiga-Y51, could be incorporated within the hydrophobic core of Aβ oligomers [22]. Our imaging mass spectrometry demonstrated the accumulation of Shiga-Y51 in the brain regions where Aβ was detected in a mouse model of AD after intravenous injection [22]. However, our previous study did not examine whether Shiga-Y51 is a feasible imaging probe for Aβ oligomers.

In this study, we investigated the in vivo detection of Aβ oligomers using Shiga-Y51 by fluorine-19 (^19^F) magnetic resonance (MR) imaging (MRI) in an APP/PS1 double transgenic mouse model of AD. Our results demonstrated significantly high levels of ^19^F-MR signals in the brain of APP/PS1 mice compared to that of wild-type mice. These findings suggest that Shiga-Y51 could be a potential imaging probe for Aβ oligomers.

## 2. Results

### 2.1. Changes in the Levels of ^19^F-NMR Signals in the Mouse Head

Shiga-Y51 is a fluorinated curcumin derivative with methyl and ethyl groups at the C4 position, which fix it as the keto form in keto-enol tautomerism (Figure 1). APPswe/PS1dE9 double transgenic (APP/PS1) mice and wild-type mice were intravenously injected with Shiga-Y51 at a dose of 200 mg/kg by continuous infusion over a 40 min period under deep anesthesia with sodium pentobarbital (50 mg/kg, i.p.). The dose and injection rate of 0.5 mg/kg/min were determined based on the previous study using another curcumin derivative Shiga-Y5 [23]. Our previous study using Shiga-Y5 indicated that the dose of 200 mg/kg was tolerated and that the higher dose of 300 mg/kg was lethal (data not shown). Furthermore, the highest level of the compound was detected when the compound was injected at 0.5 mg/kg/min via a tail vein in mice (data not shown). In this study, we preliminarily injected Shiga-Y51 at the dose of 200 mg/kg and the rate of 0.5 mg/kg/min in two mice to determine the tolerance dose in mice and found no apparent toxicity in the two mice.

In this study, mice were subjected to in vivo MR measurement under deep anesthesia with intermittent infusions of sodium pentobarbital through a polyethylene tube inserted intraperitoneally, because general inhalation anesthetic agents such as isoflurane, sevoflurane, and desflurane contain fluorine atoms, causing background noise signals. We monitored the respiratory rate and rectal temperature to determine the timing and additional dosage of sodium pentobarbital; however, infrequently, the mice woke up or died. When the mice woke up during the MR measurement, the experiment was discontinued.

**Figure 2:**
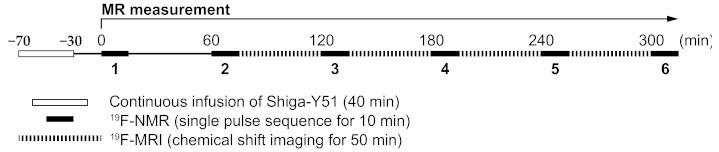
Timeline of the present study. Shiga-Y51 (200 mg/kg, i.v.) was injected by a continuous infusion over 40 min in APP/PS1 and wild-type mice under deep anesthesia. The first fluorine-19 nuclear magnetic resonance (^19^F-NMR) measurement was performed 30 min after the injection and repeated every 60 min. Fluorine-19 chemical shift imaging (^19^F-CSI) data for the fluorine-19 magnetic resonance (^19^F-MR) images were collected for 50 min every 1 h after the second ^19^F-NMR measurement.

At 30 min after the injection, ^19^F nuclear MR (^19^F-NMR) was obtained for 10 min using a 7 T MR scanner and repeated every 60 min (Figure 2). After the second ^19^F-NMR measurement, ^19^F chemical shift imaging (^19^F-CSI) data for the ^19^F-MR images were acquired for 50 min every 1 h (Figure 2).

**Figure 3:**
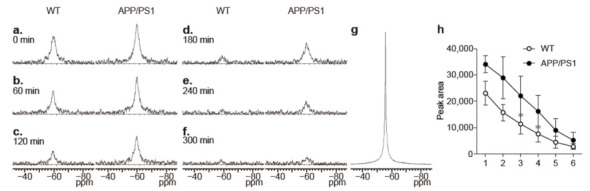
^19^F-NMR spectra from the mouse head. (**a**–**f**) Representative ^19^F-NMR spectra from the mouse heads of wild-type (WT) and APP/PS1 mice that were obtained every 60 min from 30 min after the injection. The spectra in (**a**) to (**f**) were from the measurements 1 to 6 in Figure 2, respectively. (**g**) ^19^F-NMR spectra of Shiga-Y51 dissolved in DMSO. Chemical shifts (ppm) were referenced to C_6_F_6_ at −163 ppm as an external standard. The FID signal was processed with 40 Hz line broadening similarly to CSI data. The line width of ^19^F signal in the ^19^F-NMR of Shiga-Y51 dissolved in DMSO was 177 Hz, whereas the ^19^F-NMR spectra from the mouse head showed the line width of 760 Hz. (**h**) The time course of changes in the peak area was measured in WT (*n* = 4) and APP/PS1 mice (*n* = 3). Data are presented as mean ± standard error of mean (SEM).

The ^19^F-NMR spectra obtained every 60 min demonstrated intense ^19^F signals at the first measurement and time-dependent declines in ^19^F signals in the wild-type mice (Figure 3). In contrast, higher levels of ^19^F signals were detected in APP/PS1 mice at the first ^19^F-NMR measurement and thereafter (Figure 3). However, no statistically significant differences were observed in the levels of ^19^F-NMR signals between the wild-type and APP/PS1 mice at any time points (Figure 3).

### 2.2. ^19^F-MRI

**Figure 4:**
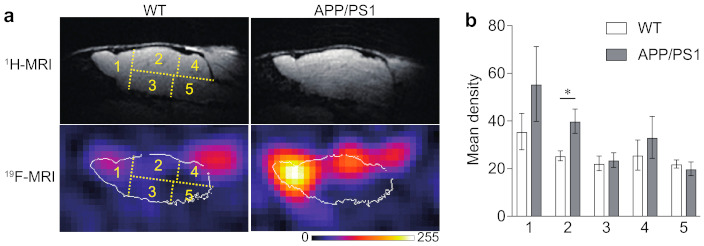
Accumulation of ^19^F signals in the brain of APP/PS1 mice. (**a**) Representative ^19^F-MR images in wild-type (WT) and APP/PS1 mice that were obtained for 50 min in the first ^19^F-CSI measurement (at 100 min after the injection). A lookup table (LUT) was used to display the ^19^F-MRI signal, and the white lines in each ^19^F-MR image indicate the outline of the brain delineated based on the corresponding ^1^H-MR images. (**b**) The levels of ^19^F signals were measured in five regions indicated in yellow color. Significantly high levels of ^19^F signals were detected in region 2 in APP/PS1 mice (*n* = 3) compared with WT mice (*n* = 4). Data are presented as mean ± SEM. * *p* < 0.05 (unpaired *t*-test).

In this study, ^19^F-CSI data were collected without a slice-selective pulse. Therefore, the ^19^F-MR images displayed whole signals covered by the coil sensitivity, including not only in the brain but also in other tissues surrounding the brain, such as eyes and ears. This experimental condition makes it difficult to analyze ^19^F signals in the olfactory bulb and the cerebellum, as they are masked by the signals in the eyes and ears, where fluorinated curcumin derivatives could be highly accumulated, because of high hydrophobicity. Hence, we analyzed ^19^F signals separately in five regions (Figure 4). Region 1 includes the olfactory bulbs and a part of the cerebral cortex wherein the signals in the eyes overlap. Regions 2 and 3 include the upper and lower parts of the brain, respectively. Region 4 includes the cerebellum wherein the signals in the ears overlap, and region 5 includes the brain stem.

The wild-type (WT) mice displayed relatively high ^19^F signals in regions 1 and 4 in the first ^19^F-CSI measurement obtained for 50 min from 100 min after injection with Shiga-Y51 (Figure 4). The APP/PS1 mice displayed intense ^19^F signals in region 2, in addition to regions 1 and 4 (Figure 4). The ^19^F signals in ^19^F-MR images were then decreased in a time-dependent manner in both WT and APP/PS1 mice (data not shown). The levels of ^19^F signals in each region in the first ^19^F-CSI measurement exhibited a significant difference in region 2 between the WT and APP/PS1 mice (*p* < 0.05; Figure 4). However, there were no significant differences in the levels of ^19^F signals in other regions.

### 2.3. Levels of Aβ in the Brain of APP/PS1 Mice

**Figure 5:**
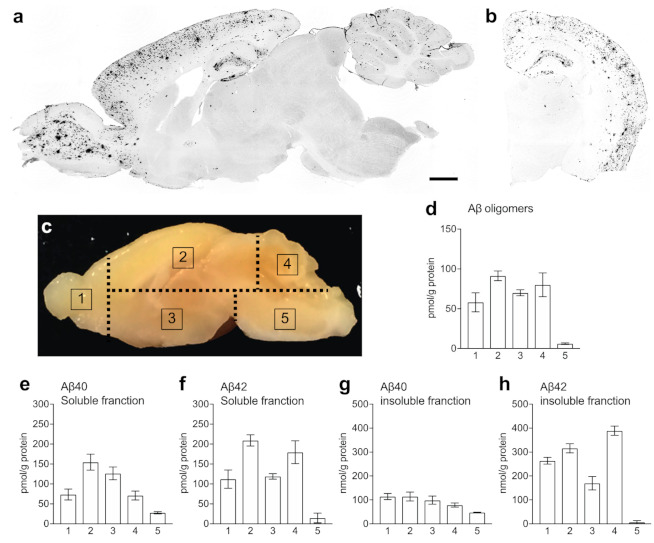
The levels of amyloid β (Aβ) in APP/PS1 mice. (**a,b**) Representative photographs showing immunohistochemistry for Aβ in the sagittal (**a**) and coronal (**b**) sections of APP/PS1 mouse brain. (**c**–**h**) The brains were cut into five pieces according to the dashed lines in (**c**), and the levels of Aβ oligomers (**d**) in the soluble fraction and Aβ40 (**e**,**g**) and Aβ42 (**f**,**h**) in the soluble (**e**,**f**) and insoluble (**g**,**h**) fractions were measured in each region. Data are presented as mean ± SEM. Scale bar: 1 mm.

Aβ accumulation was detected in the olfactory bulb, cerebral cortex, hippocampus, and cerebellum of APP/PS1 mice (Figure 5). To validate the ^19^F-MRI results, we measured the levels of soluble Aβ oligomers in the five regions using a commercially available enzyme-linked immunosorbent assay (ELISA) kit with the same monoclonal antibody specific to the N-terminal region of human Aβ (clone 82E1) for both capture and detection. This ELISA kit can detect human Aβ molecules that bind to 82E1 antibody with two or more epitopes. The measurement result is calculated in molarity as a relative value standardized on the dimers of Aβ (1–16 residues). Our results revealed the highest accumulation of soluble Aβ oligomers in region 2, followed by regions 4, 3, and 1. Region 5 showed very low levels of soluble Aβ oligomers. Then, we measured the levels of soluble and insoluble Aβ in the five regions. The highest levels of soluble Aβ40 were detected in region 2, followed by regions 3, 4, 1, and 5 (Figure 5). Moreover, the pattern of the accumulation levels of soluble Aβ42 in the five regions was similar to that of soluble Aβ oligomers, i.e., the highest level of soluble Aβ42 was detected in region 2, followed by regions 4, 3, and 1. The levels of insoluble Aβ, which reflected the Aβ plaques consisting of insoluble Aβ fibrils, were the highest in regions 1 and 2, followed by regions 3, 4, and 5 in insoluble Aβ40, and in region 4, followed by regions 2, 1, 3, and 5 in insoluble Aβ42.

## 3. Discussion

We explored whether Shiga-Y51 detected Aβ oligomers in the brain of APP/PS1 mice using ^19^F-MRI with a 7 T MR scanner. Our results revealed the presence of significantly high levels of ^19^F signals in the brain region, including the upper forebrain, of APP/PS1 mice, compared with the levels found in brains of WT mice. Moreover, the highest levels of Aβ oligomers were detected in the upper forebrain of APP/PS1 mice. Our study findings suggested that ^19^F-MRI using Shiga-Y51 detected Aβ oligomers in the in vivo brain of APP/PS1 mice.

Aβ can easily polymerize with β-sheeted structures and form insoluble Aβ fibrils, which accumulate in deposits known as senile plaques. Aβ can also form a different type of aggregates, the so-called Aβ oligomers, which are small, soluble, and diffusible enough to readily pass through the brain parenchyma to induce synaptic dysfunction and memory impairment [24,25,26,27]. Our previous study demonstrated that curcumin derivatives with keto-enol tautomerism bound to Aβ fibrils and oligomers [28,29]. In contrast, a keto form of curcumin derivatives such as Shiga-Y51 exhibited high selectivity to Aβ oligomers in vitro [22]. In this study, we used the APP/PS1 mouse model to verify the in vivo detection of Aβ oligomers using Shiga-Y51 by ^19^F-MRI. This mouse model suffered from the accumulation of both Aβ oligomers and fibrils in the brain regions where ^19^F signals were detected in ^19^F-MRI. Therefore, although we expected Shiga-Y51 to bind selectively to Aβ oligomers in the in vivo brain as well, there could be the possibility of its off-target binding to Aβ fibrils in the APP/PS1 mouse brain. However, for detecting the signals in MRI, it is necessary to ensure that the free mobility of NMR nuclei is not inhibited. When the free mobility of NMR nuclei is inhibited, the NMR signals would be dramatically reduced due to shortening of T_2_ and broadening of the NMR signal [30]. Based on this knowledge, the ^19^F signals of Shiga-Y51 that possibly bind to Aβ fibrils could not be detectable in the present study. In contrast, Shiga-Y51 bound to Aβ oligomers would retain its mobility because of the free mobility of soluble Aβ oligomers. Hence, we concluded that ^19^F-MRI using Shiga-Y51 in the present study would detect soluble Aβ oligomers but not insoluble Aβ fibrils in the brain of APP/PS1 mice. In addition, highly hydrophobic fluorine probes could be trapped by the lipid components of the brain such as myelin, causing shortening of T_2_. Our preliminary study suggests that FID-type CSI data acquisition using a surface coil was suitable for the detection of the relatively short T_2_ of fluorine probes in the brain (data not shown), rather than echo signal of rapid acquisition with relaxation enhancement (RARE) used by others [31]. Although further validation and improvement of sensitivity are needed, MRI using molecular imaging probes would have great potential in detecting the soluble oligomers of proteins that also form insoluble fibrillar aggregates in the brain in neurogenerative diseases, such as Aβ, tau, α-synuclein, and TDP-43.

This study showed a significant accumulation of ^19^F signals in brain region 2 in APP/PS1 mice compared with WT mice. Region 2, consisting of the upper forebrain, including the cerebral cortex and hippocampus, showed the highest level of Aβ oligomers in ELISA measurements. Furthermore, no obvious background noise was detected in region 2, and the position being closer to the coil than region 3 would be preferable for highly sensitive detection. These results suggest that the levels of 19F signals could reflect the level of Aβ oligomers in brain region 2 in APP/PS1 mice. In contrast, the levels of ^19^F signals in region 5 were not much different from those in regions 1 to 4 where at least a 5-fold increase in levels of Aβ oligomers was detected in ELISA measurements. Sensitivity and resolution would be improved to reflect the actual difference in the levels of Aβ oligomers in each region.

In this study, there were no significant differences detected in ^19^F-NMR signals between the WT and APP/PS1 mice, although higher levels of ^19^F signals were detected in APP/PS1 mice at all time points. In this study, we used a circular-type surface coil measuring 1.6 cm in diameter and collected whole signals covered by the coil sensitivity in the ^19^F-MR measurement. Consequently, ^19^F signals could be detected not only in the brain but also in other tissues surrounding the brain, such as eyes and ears. As shown in Figure 4, high levels of ^19^F signals were detected in the eyes and ears of WT mice, which also overlapped the brain. Accordingly, the levels of ^19^F-NMR signals that consisted of the signals not only in the brain but also in the eyes and ears would not reflect the levels of Shiga-Y51 accumulated in the brain.

Several positron emission tomography tracers are clinically available, such as PiB, flutemetamol, florbetaben, and florbetapir, which have been developed for imaging Aβ fibrils in the brain [32]. However, there is a lack of a clinically available method for detecting Aβ oligomers, although several attempts using NIR fluorescence imaging in mouse models of AD have been successful, including BoDipy-Oligomer (BD-Oligo) [17], F-SLOH [18], CRANAD-102 [19], PTO-29 [20], and DCM-AN [21]. Shiga-Y51 is a novel fluorinated curcumin derivative with a fixed keto form, which is a completely different structure from that of compounds targeting Aβ oligomers previously reported. Moreover, compared with NIR fluorescence imaging, MRI has the advantage of the imaging depth. Hence, it is probable that ^19^F-MRI using Shiga-Y51 would be a more feasible technique to detect Aβ oligomers in the human brain.

^19^F-MRI would prove to be a highly sensitive, readily available, low-background, and cost-effective approach once a suitable high-quality probe has been developed, including FSB [31], Shiga-Y5 [23], Shiga-X22 [33], and ShigaX35 [34], due to the following advantages: the MR sensitivity of ^19^F is relatively high compared with various nuclei other than ^1^H (^1^H, 100%; ^19^F, 83%; ^31^P, 6.6%; ^13^C, 1.6%). No detectable fluorine atoms exist in biological tissues (fluorine in bones and teeth are not appropriate for ^19^F MRI relaxation time), which could result in low endogenous background noise. Furthermore, the ^19^F atom is a nonradioactive isotope comprising 100% of naturally abundant fluorine. However, there were some limitations in the application of ^19^F-MRI using Shiga-Y51 for imaging Aβ oligomers. First, since strong unwanted signals were also detected even in the WT mouse brain, in which the signals in the eyes and ears overlapped (the signals may not be a problem in human), it is necessary to improve the accumulation of Shiga-Y51 in these tissues through modifications to the probe structure. Second, the present ^19^F-MRI methodology requires a 7 T MR scanner; however, the 7 T MR scanner is not a common imaging modality at the moment. Third, the method of general anesthesia during long MR measurement should be improved. Although we have no evidence of whether a significant dose of anesthesia would affect the results, it may be possible that an additional dose of anesthesia between mice would affect the results, because anesthesia depth could affect general parameters such as blood circulation, respiration, and body temperature. Finally, it is important to improve the sensitivity for safety reasons, because in this study, quite a high dose of probe (200 mg/kg) (which may be near the value of LD_50_) was used for ^19^F-MRI. In an in vitro study, increasing the total number of fluorine atoms in the probe enabled us to detect higher NMR signals. However, the hydrophobicity of fluorine probes increases with the number of fluorine atoms because of the hydrophobic property of fluorine atoms. This problem would cause low water solubility and interaction with the lipid components of the brain, such as myelin [30,33]. Thus, increasing the number of fluorine atoms in the probe would not be an appropriate way to develop a highly sensitive probe. In addition, improvement of water solubility of Shiga-Y51 is needed to prepare the injection solution without a detergent.

In conclusion, understanding the accumulation of Aβ oligomers in the brain would provide novel insights into the etiology, diagnosis, and treatment of AD. The results of this study suggest that ^19^F-MRI using Shiga-Y51 with a 7 T MR scanner is a powerful tool for imaging Aβ oligomers in the brain. Although further attempts are required for clinical application, Shiga-Y51 could be a first-generation imaging probe targeting Aβ oligomers in the brain.

## 4. Materials and Methods

### 4.1. Synthesis of Shiga-Y51

Shiga-Y51, namely, 1,7-bis(4′-hydroxy-3′-trifluoromethoxy)phenyl-4-ethyl-4-methyl-1,6-heptadiene-3,5-dione, was synthesized as previously described [22].

### 4.2. Animals

All animal experiments were conducted according to the National Institutes of Health Guide for the Care and Use of Laboratory Animals and were approved by the Animal Care and Use Committee at the Shiga University of Medical Science (Number 2018-2-12).

APP/PS1 mice with a C57BL/6 background (Jackson Laboratory, Bar Harbor, ME, USA), expressing a chimeric mouse/human amyloid precursor protein with the K594N and M595L mutations linked to Swedish familial AD (Mo/HuAPP695swe) and human PS1, carrying the exon 9 deletion associated with familial AD [35], were used in this study. The mice were housed in standard laboratory cages at 23 °C with free access to water and food in an SPF animal facility and maintained with a 12 h light/dark cycle with the lights on from 8:00 a.m. to 20:00 p.m.

Female APP/PS1 mice (*n* = 4) aged 14.5–16.5 months and female WT mice (*n* = 3) aged 11.5–17 months were used for the MRI study. For ELISA measurements, female APP/PS1 mice aged 9 and 15 months (*n* = 3 each) were used.

### 4.3. MRI

A 7 T horizontal-bore MR scanner (Unity Inova; Agilent Technologies, Santa Clara, CA, USA) was used in this study. A custom-built circular-type surface coil (Takashima Seisakusho, Tokyo, Japan) measuring 1.6 cm in diameter and tuned to both the ^1^H and ^19^F frequencies (300 and 282 MHz, respectively) was used to collect the data. This RF coil had no fluorine-containing materials, which cause instrument noise [36].

^1^H- and ^19^F-MR images were acquired as previously described [23,33,34]. Briefly, to obtain ^1^H-MR images of the mouse brain, a gradient-echo sequence was used with 150 ms repetition time (TR), 3 ms echo time, 60° flip angle, 1.5 mm slice thickness, 24 mm × 24 mm field of view, and 128 × 128 resolution.

#### 4.3.1. ^19^F-NMR Spectrum

A nonlocalized ^19^F-NMR spectrum was obtained from the whole head using a single pulse sequence with 8192 data points, 40,000 Hz spectral width, 1 s TR, and 600 acquisitions (for 10 min).

#### 4.3.2. ^19^F-CSI

To obtain ^19^F-MR images, free induction decay data of ^19^F-CSI were collected with a 40,000 Hz spectral width, 24 mm × 24 mm field of view in the sagittal plane, 1 s TR, 200 µs phase-encoding time, and 68 acquisitions for each central 44 phase-encoding step of 8 × 8 steps. For the residual 20 phase-encoding steps in the periphery of k-space, zero data were used. The total acquisition time for one data set was 50 min. As we did not use a slice-selective pulse, whole signals covered by the coil sensitivity were acquired. The raw data were processed by 3D-Fourier transformation with 40 Hz line broadening and zero filling and finally converted into 32 × 32 spectral data sets.

#### 4.3.3. Procedure of MR Measurement

Shiga-Y51 was dissolved at a concentration of 10 mg/mL in saline containing 10% Cremophor EL. Under anesthesia using sodium pentobarbital (50 mg/kg, i.p.), mice were intravenously injected at a dose of 200 mg/kg via the tail vein over a 40 min period by continuous infusion at a rate of 0.5 mL/kg/min. Immediately after the injection, the mice, whose heads were set on the RF coil, were placed in the MR scanner, and the ^1^H-MR images were acquired. ^19^F-NMR were measured for 10 min at 30 min after the injection and repeated every 60 min. ^19^F-CSI data for the ^19^F-MR images were collected for 50 min every 1 h after the second ^19^F-NMR measurement (Figure 2).

General anesthesia was maintained with intermittent infusion of sodium pentobarbital through a polyethylene tube inserted intraperitoneally. Any additional dosage of sodium pentobarbital required for maintaining anesthesia was determined by monitoring the respiratory rate. The animals were warmed with an air drier, and the rectal temperature was monitored throughout the experiments.

### 4.4. Immunohistochemistry

Mice were sacrificed under deep anesthesia with sodium pentobarbital (200 mg/kg, i.p.). The brain was quickly removed from each mouse. One brain hemisphere was post-fixed in 4% paraformaldehyde for 24 h at 4 °C, and the other hemisphere was dissected into five pieces as shown in Figure 5 and was stored at −80 °C. After 24 h post-fixation, the brain hemisphere was immersed in 0.1 M phosphate buffer (pH 7.4) containing 15% sucrose and 0.1% sodium azide for at least 2 days for cryoprotection and subsequently cut into 20 µm sections in a cryostat.

Immunohistochemistry for detecting Aβ was performed as previously described [37,38]. Briefly, the free-floating brain sections in the sagittal plane were treated with 0.3% hydrogen peroxide in 0.1 M phosphate-buffered saline (PBS) containing 0.3% Triton X-100 (PBS-T, pH 7.4) to eliminate endogenous peroxidase activity. After several washes, the sections were treated with 2% bovine serum albumin (BSA) in PBS-T for 30 min at room temperature to block nonspecific protein binding. The sections were then incubated with rabbit polyclonal antibodies against the N-terminal region of human Aβ (1:500; Immuno-Biological Laboratories, Fujioka, Japan) in PBS-T containing 0.2% BSA for 24 h at 4 °C, followed by incubation with biotinylated anti-rabbit IgG (1:1000; Vector Laboratories, Burlingame, CA, USA) for 1 h at room temperature. The sections were then incubated with the avidin–biotin–peroxidase complex (Vectastatin ABC Elite kit, 1:3000; Vector Laboratories) for 1 h at room temperature. All sections were washed several times with PBS-T between each step, and labeling was detected using 3,3ʹ-diaminobenzidine (DAB; Dojindo Laboratories, Kumamoto, Japan), with nickel ammonium, which yielded a dark blue color. The sections were then mounted on glass slides and coverslipped with Entellan (Merck, Darmstadt, Germany).

### 4.5. ELISA

Brain tissue extracts were prepared for ELISA measurements as previously described [37,38]. Briefly, frozen brain tissues were homogenized on ice in Tris-buffered saline (TBS; 25 mM Tris-HCl, 150 mM NaCl, pH 7.4) (1-mL/150-mg wet weight) containing a protease inhibitor cocktail (cOmplete Mini; Roche Diagnostics, Mannheim, Germany), sonicated, and then centrifuged at 104,300× *g* for 60 min at 4 °C. The supernatant was collected as a TBS-soluble fraction, and the remaining pellet was then resuspended in 70% formic acid in water, sonicated, and then centrifuged at 104,300× *g* for 60 min at 4 °C. The supernatant was recovered and neutralized with a 20 fold dilution in 1 M Tris base. The protein concentration for each sample was determined using a Bio-Rad protein assay kit (Bio-Rad Laboratories, Hercules, CA, USA).

The levels of Aβ40 and Aβ42 were measured using a commercially available ELISA kit (294-64701 and 290-62601, respectively; Wako Pure Chemicals, Osaka, Japan), according to the manufacturer’s instructions. To detect Aβ oligomer species, an ELISA kit with the same monoclonal antibody specific to the N-terminal region of human Aβ (clone 82E1) for both capture and detection (27725; Immuno-Biological Laboratories, Fujioka, Japan) was used. The absorbance in each well was measured at 450 nm using a microplate reader (Infinite M200; Tecan, Männedorf, Switzerland).

### 4.6. Statistical Analysis

Data are presented as mean ± standard error of mean (SEM). The statistical significance between wild-type control mice and APP/PS1 mice was analyzed by an unpaired *t*-test using GraphPad Prism 7 (GraphPad Software, La Jolla, CA, USA).

## 5. Patents

Shiga University of Medical Science has submitted a Japanese patent application (JP2020-033405) on Shiga-Y51, with D.Y., H.T. and I.T. named as the inventors.

## Data Availability

The data presented in this study are available on request from the corresponding author. The data are not publicly available as the data also form a part of an ongoing study.

## Sample Availability

Samples of the compounds are available from the authors.

## References

[1] Glenner G.G., Wong C.W. (1984). Alzheimer’s disease: Initial report of the purification and characterization of a novel cerebrovascular amyloid protein. Biochem. Biophys. Res. Commun..

[2] Hardy J.A., Higgins G.A. (1992). Higgins Alzheimer’s Disease: The Amyloid Cascade Hypothesis. Science.

[3] Hardy J., Selkoe D.J. (2002). The Amyloid Hypothesis of Alzheimer ’ s Disease: Progress and Problems on the Road to Therapeutics. Science.

[4] Selkoe D.J., Hardy J. (2016). The amyloid hypothesis of Alzheimer’s disease at 25 years. EMBO Mol. Med..

[5] Tomiyama T., Nagata T., Shimada H., Teraoka R., Fukushima A., Kanemitsu H., Takuma H., Kuwano R., Imagawa M., Ataka S. (2008). A new amyloid β variant favoring oligomerization in Alzheimer’s-type dementia. Ann. Neurol..

[6] Nilsberth C., Westlind-Danielsson A., Eckman C.B., Condron M.M., Axelman K., Forsell C., Stenh C., Luthman J., Teplow D.B., Younkin S.G. (2001). The “Arctic” APP mutation (E693G) causes Alzheimer’s disease by enhanced Aβ protofibril formation. Nat. Neurosci..

[7] Kamino K., Orr H.T., Payami H., Wijsman E.M., Alonso M.E., Pulst S.M., Anderson L., O’dahl S., Nemens E., White J.A. (1992). Linkage and mutational analysis of familial Alzheimer disease kindreds for the APP gene region. Am. J. Hum. Genet..

[8] Cline E.N., Bicca M.A., Viola K.L., Klein W.L. (2018). The Amyloid-β Oligomer Hypothesis: Beginning of the Third Decade. J. Alzheimer’s Dis..

[9] Ono K., Tsuji M. (2020). Protofibrils of amyloid-β are important targets of a disease-modifying approach for alzheimer’s disease. Int. J. Mol. Sci..

[10] Huang Y.R., Liu R.T. (2020). The toxicity and polymorphism of β-amyloid oligomers. Int. J. Mol. Sci..

[11] Yang J., Perrett S. (2021). Single Molecule Characterization of Amyloid Oligomers. Molecules.

[12] Krishnadas N., Villemagne V.L., Doré V., Rowe C.C. (2021). Advances in Brain Amyloid Imaging. Semin. Nucl. Med..

[13] Hilt S., Tang T., Walton J.H., Budamagunta M., Maezawa I., Kálai T., Hideg K., Singh V., Wulff H., Gong Q. (2016). A Metal-Free Method for Producing MRI Contrast at Amyloid-β. J. Alzheimer’s Dis..

[14] Yeo S.K., Shepelytskyi Y., Grynko V., Albert M.S. (2020). Molecular imaging of fluorinated probes for tau protein and amyloid-β detection. Molecules.

[15] Santin M.D., Vandenberghe M.E., Herard A.S., Pradier L., Cohen C., Debeir T., Delzescaux T., Rooney T., Dhenain M. (2016). In vivo detection of amyloid plaques by gadolinium-stained MRI can be used to demonstrate the efficacy of an anti-amyloid immunotherapy. Front. Aging Neurosci..

[16] Adlard P.A., Tran B.A., Finkelstein D.I., Desmond P.M., Johnston L.A., Bush A.I., Egan G.F. (2014). A review of β-amyloid neuroimaging in Alzheimer’s disease. Front. Neurosci..

[17] Teoh C.L., Su D., Sahu S., Yun S.W., Drummond E., Prelli F., Lim S., Cho S., Ham S., Wisniewski T. (2015). Chemical Fluorescent Probe for Detection of Aβ Oligomers. J. Am. Chem. Soc..

[18] Li Y., Xu D., Sun A., Ho S.L., Poon C.Y., Chan H.N., Ng O.T.W., Yung K.K.L., Yan H., Li H.W. (2017). Fluoro-substituted cyanine for reliable: In vivo labelling of amyloid-β oligomers and neuroprotection against amyloid-β induced toxicity. Chem. Sci..

[19] Li Y., Yang J., Liu H., Yang J., Du L., Feng H., Tian Y., Cao J., Ran C. (2017). Tuning the stereo-hindrance of a curcumin scaffold for the selective imaging of the soluble forms of amyloid beta species. Chem. Sci..

[20] Yang J., Zeng F., Li X., Ran C., Xu Y., Li Y. (2020). Highly specific detection of Aβ oligomers in early Alzheimer’s disease by a near-infrared fluorescent probe with a “v-shaped” spatial conformation. Chem. Commun..

[21] Lv G., Sun A., Wang M., Wei P., Li R., Yi T. (2020). A novel near-infrared fluorescent probe for detection of early-stage Aβ protofibrils in Alzheimer’s disease. Chem. Commun..

[22] Yanagisawa D., Kato T., Taguchi H., Shirai N., Hirao K., Sogabe T., Tomiyama T., Gamo K., Hirahara Y., Kitada M. (2021). Keto form of curcumin derivatives strongly binds to Aβ oligomers but not fibrils. Biomaterials.

[23] Yanagisawa D., Amatsubo T., Morikawa S., Taguchi H., Urushitani M., Shirai N., Hirao K., Shiino A., Inubushi T., Tooyama I. (2011). In vivo detection of amyloid β deposition using ^19^F magnetic resonance imaging with a ^19^F-containing curcumin derivative in a mouse model of Alzheimer’s disease. Neuroscience.

[24] Gong Y., Chang L., Viola K.L., Lacor P.N., Lambert M.P., Finch C.E., Krafft G.A., Klein W.L. (2003). Alzheimer’s disease-affected brain: Presence of oligomeric Aβ ligands (ADDLs) suggests a molecular basis for reversible memory loss. Proc. Natl. Acad. Sci. USA.

[25] Lesné S., Ming T.K., Kotilinek L., Kayed R., Glabe C.G., Yang A., Gallagher M., Ashe K.H. (2006). A specific amyloid-β protein assembly in the brain impairs memory. Nature.

[26] Walsh D.M., Klyubin I., Fadeeva J.V., Cullen W.K., Anwyl R., Wolfe M.S., Rowan M.J., Selkoe D.J. (2002). Naturally secreted oligomers of amyloid β protein potently inhibit hippocampal long-term potentiation in vivo. Nature.

[27] Shankar G.M., Li S., Mehta T.H., Garcia-Munoz A., Shepardson N.E., Smith I., Brett F.M., Farrell M.A., Rowan M.J., Lemere C.A. (2008). Amyloid-β protein dimers isolated directly from Alzheimer’s brains impair synaptic plasticity and memory. Nat. Med..

[28] Yanagisawa D., Taguchi H., Yamamoto A., Shirai N., Hirao K., Tooyama I. (2011). Curcuminoid binds to amyloid-β_1-42_ oligomer and fibril. J. Alzheimer’s Dis..

[29] Yanagisawa D., Shirai N., Amatsubo T., Taguchi H., Hirao K., Urushitani M., Morikawa S., Inubushi T., Kato M., Kato F. (2010). Relationship between the tautomeric structures of curcumin derivatives and their Aβ-binding activities in the context of therapies for Alzheimer’s disease. Biomaterials.

[30] Amatsubo T., Morikawa S., Inubushi T., Urushitani M., Taguchi H., Shirai N., Hirao K., Kato M., Morino K., Kimura H. (2009). Trifluoromethoxy-benzylated ligands improve amyloid detection in the brain using ^19^F magnetic resonance imaging. Neurosci. Res..

[31] Higuchi M., Iwata N., Matsuba Y., Sato K., Sasamoto K., Saido T.C. (2005). ^19^F and ^1^H MRI detection of amyloid β plaques in vivo. Nat. Neurosci..

[32] Zetterberg H., Bendlin B.B. (2020). Biomarkers for Alzheimer’s disease—Preparing for a new era of disease-modifying therapies. Mol. Psychiatry.

[33] Yanagisawa D., Taguchi H., Ibrahim N.F., Morikawa S., Shiino A., Inubushi T., Hirao K., Shirai N., Sogabe T., Tooyama I. (2014). Preferred features of a fluorine-19 MRI probe for amyloid detection in the brain. J. Alzheimer’s Dis..

[34] Yanagisawa D., Ibrahim N.F.N.F., Taguchi H., Morikawa S., Kato T., Hirao K., Shirai N., Sogabe T., Tooyama I. (2018). Fluorine-19 magnetic resonance imaging probe for the detection of tau pathology in female rTg4510 mice. J. Neurosci. Res..

[35] Jankowsky J.L., Fadale D.J., Anderson J., Xu G.M., Gonzales V., Jenkins N.A., Copeland N.G., Lee M.K., Younkin L.H., Wagner S.L. (2004). Mutant presenilins specifically elevate the levels of the 42 residue β-amyloid peptide in vivo: Evidence for augmentation of a 42-specific γ secretase. Hum. Mol. Genet..

[36] Amatsubo T., Yanagisawa D., Morikawa S., Taguchi H., Tooyama I. (2010). Amyloid imaging using high-field magnetic resonance. Magn. Reson. Med. Sci..

[37] Yanagisawa D., Ibrahim N.F., Taguchi H., Morikawa S., Hirao K., Shirai N., Sogabe T., Tooyama I. (2015). Curcumin derivative with the substitution at C-4 position, but not curcumin, is effective against amyloid pathology in APP/PS1 mice. Neurobiol. Aging.

[38] Ibrahim N.F., Yanagisawa D., Durani L.W., Hamezah H.S., Damanhuri H.A., Wan Ngah W.Z., Tsuji M., Kiuchi Y., Ono K., Tooyama I. (2017). Tocotrienol-Rich Fraction Modulates Amyloid Pathology and Improves Cognitive Function in AβPP/PS1 Mice. J. Alzheimer’s Dis..

